# Association Between Family Dysfunction and Risk for Eating Disorders in Adolescents

**DOI:** 10.3390/jcm15051726

**Published:** 2026-02-25

**Authors:** Mario J. Valladares-Garrido, Camila I. Medina-Quispe, Darwin A. León-Figueroa, María Julia Cómina-Tamayo, Luz A. Aguilar-Manay, Jassmin Santin Vásquez, Danai Valladares-Garrido, César J. Pereira-Victorio, Víctor J. Vera-Ponce, Oriana Rivera-Lozada

**Affiliations:** 1 Escuela de Medicina Humana, Universidad Señor de Sipán, Chiclayo 14007, Peru; riveraoriana@uss.edu.pe; 2 Departamento de Ciencias Médicas, Facultad de Ciencias de la Salud, Universidad de Castilla-La Mancha, 13071 Ciudad Real, Spain; 3 Escuela Profesional de Medicina Humana, Universidad Nacional de San Cristóbal de Huamanga, Ayacucho 05003, Peru; camila.2468.2468.2468@gmail.com; 4 Facultad de Medicina Humana, Universidad de San Martín de Porres, Chiclayo 14001, Peru; dalefi19@gmail.com (D.A.L.-F.); luz_aguilar2@usmp.pe (L.A.A.-M.); jassmin_santin@usmp.pe (J.S.V.); 5 Escuela de Medicina Humana, Universidad Peruana Cayetano Heredia, Lima 15102, Peru; maria.comina@upch.pe; 6 Escuela de Medicina, Universidad César Vallejo, Trujillo 13001, Peru; danai.paola@gmail.com; 7 Oficina de Salud Ocupacional, Hospital Santa Rosa, Piura 20001, Peru; 8 EpiHealth Research Center for Epidemiology and Public Health, Lima 15074, Peru; 9 Facultad de Medicina, Universidad Continental, Lima 15046, Peru; pereira.victorio.cj@gmail.com; 10 Facultad de Medicina (FAMED), Universidad Nacional Toribio Rodríguez de Mendoza de Amazonas (UNTRM), Chachapoyas 15046, Peru; victor_jvp@hotmail.com

**Keywords:** adolescents, COVID-19 pandemic, cross-sectional studies, eating disorders, family dysfunction

## Abstract

**Background/Objectives**: Risk for eating disorders (ED risk) in adolescents is strongly influenced by psychosocial factors, particularly family dysfunction. The COVID-19 pandemic may have intensified these risks by disrupting family dynamics and increasing stress exposure. This study aimed to examine the association between family dysfunction and ED risk among school adolescents in Lambayeque, Peru, in the post-pandemic context. **Methods**: An analytical cross-sectional study including 1219 students from five schools (September–December 2022) was conducted. ED risk was screened using the SCOFF questionnaire (≥2), and family functioning was assessed with the Family APGAR (functional, mild, moderate, or severe dysfunction). Additional sociodemographic, behavioral, and mental health variables were analyzed. Adjusted prevalence ratios (PRs) were estimated using Poisson regression with robust variance clustered by school. **Results**: The ED risk in adolescents was 39.3% (95% CI: 36.5–42.1). Moderate family dysfunction was reported in 10.0% and severe dysfunction in 29.8% of participants. In the multivariable model, moderate family dysfunction was associated with higher ED risk (PR = 1.11). Other factors associated with higher risk included obesity (PR = 1.17), family history of mental illness (PR = 1.18), course failure (PR = 1.18), alcohol consumption ≥4 times per week (PR = 1.75), and having a family member hospitalized due to COVID-19 (PR = 1.14). Protective associations were found for male sex, frequent contact with friends, higher resilience, and living in peri-urban areas. **Conclusions**: Moderate family dysfunction was associated with an increased at ED risk in adolescents. These findings highlight the importance of school-based screening, family-centered interventions, and resilience promotion in adolescent mental health strategies. Given the cross-sectional design, causal relationships cannot be inferred.

## 1. Introduction

Risk for eating disorders (ED risk) in adolescents has been linked to multiple psychosocial factors, including family dysfunction [1]. Evidence suggests that a conflictive or dysfunctional family environment can increase vulnerability to ED risk by affecting emotional regulation, self-esteem, and body image among adolescents [2]. Given the increasing burden of ED-related behaviors and their psychological consequences, clarifying family determinants associated with ED risk remains a public health priority.

At the international level, clinically diagnosed ED prevalence in adolescents remains low (0.9% in Australia, 0.89% in Iran, 1.5% in Germany, 2.7% in the United States, and 8.7% in Norway) [3,4,5,6,7]. In contrast, screening-based ED risk is substantially higher, ranging from 8.3% in Chile to 1.6% in Mexico, 3% in Bolivia, and 9.7% in Brazilian students [8,9,10,11]. In Peru, studies consistently report ED risk rather than clinical diagnoses, with proportions ranging from 12.5% to 47.6% across school and university settings [12,13,14,15]. This variability likely reflects differences in instruments, cut-off points, population characteristics, and timing of data collection (pre- vs. post-pandemic).

Family functioning is a key determinant of adolescent emotional health. Dysfunctional environments—characterized by low cohesion, poor communication, or conflict—have been linked to greater ED risk in studies across Italy [16]; Turkey [17]; Hungary [18]; Spain [19]; the United States [20]; and several Latin American countries including Colombia, Mexico, Venezuela, and Peru [21,22,23,24,25]. Across these contexts, poorer family functioning consistently correlates with disordered eating, dieting behaviors, and ED-related symptoms.

Despite extensive literature supporting these associations, several methodological limitations remain. Many prior studies failed to adjust for potential confounders such as alcohol consumption, social media use, or prior mental health conditions [17,18,26], potentially biasing estimates. Moreover, some relied on small samples with limited statistical power—often fewer than 30 families—or used only bivariate analyses without controlling for covariates [27,28]. Additionally, few studies have evaluated these associations among Peruvian school adolescents in the post-COVID-19 context, despite the pandemic’s profound psychosocial impact on families and youth.

### The Present Study

To address these gaps, the present study aims to evaluate the association between family dysfunction and ED risk among school adolescents in the Lambayeque region of Peru during the post-pandemic period. By using validated instruments (SCOFF and Family APGAR), adjusting for a broad set of psychosocial, academic, behavioral, and mental health variables, and applying robust statistical methods, this study provides updated, population-based evidence on the role of family functioning in adolescent ED risk within a Latin American post-COVID-19 context.

## 2. Materials and Methods

### 2.1. Study Design

Analytical cross-sectional study based on the secondary analysis of data obtained from adolescent students attending five secondary-level educational institutions in the Lambayeque region, Peru. Data collection was carried out between September and December 2022, in the context of the fifth wave of the COVID-19 pandemic.

The present study aimed to evaluate the association between family dysfunction and ED risk in adolescents. The data were obtained from a primary database whose original objective was to analyze the relationship between acne and mental health outcomes.

### 2.2. Population and Sample

The study population consisted of 1972 adolescents enrolled in the 2022 academic year in five secondary-level schools located in the Lambayeque region, northern Peru.

The school selection process was non-probabilistic and based on convenience, considering institutions that met the following criteria: accessibility, administrative authorization, and willingness to participate in the broader research project. Within each selected school, all eligible students were invited to participate.

In the primary study, adolescents were included if they attended classes regularly, completed the essential research questionnaires, and provided informed assent. Those whose parents did not authorize their participation through informed consent were excluded. The final response rate of the primary study was 72.8% (n = 1436). For the present secondary analysis, a new data cleaning process was performed, excluding 217 records with incomplete responses in the questionnaires of interest, specifically in the family dysfunction scale and the ED risk questionnaire. As a result, the final adjusted response rate for this study was 61.8% (n = 1219).

A statistical power calculation was performed to assess the study’s ability to detect differences between family dysfunction and ED risk in adolescents. For this calculation, the proportion of ED risk in the group without family dysfunction (p_1_ = 0.34) and in the group with family dysfunction (p_2_ = 0.43) was used, along with their respective sample sizes (n_1_ = 493 for adolescents without family dysfunction and n_2_ = 726 for adolescents with family dysfunction). A statistical power of 88.7% was obtained, indicating that the study had a high capacity to detect significant differences between the groups compared.

### 2.3. Procedures

Data collection was carried out between September and December 2022 in five secondary-level educational institutions in the Lambayeque region, Peru. A self-administered digital questionnaire, developed on the REDCap platform, was applied, which included sociodemographic variables, mental health history, family dysfunction, and ED risk.

Before implementation, a pilot test was conducted in two schools (n = 60 students) to assess comprehension, timing, and technical performance of the digital format. Minor adjustments were made to improve clarity and usability based on student feedback.

The final version was administered by a trained field team consisting of medical researchers with prior experience in adolescent mental health assessments. They received standardized training on data collection protocols, ethical considerations, and procedures to ensure uniform administration across all schools.

Before the implementation of the study, meetings were held with the authorities of each institution to coordinate logistical aspects and ensure optimal conditions for administering the questionnaire. Participants were informed about the purpose of the research, and the voluntary and confidential nature of their participation was emphasized. To comply with ethical principles, informed consent was obtained in advance from parents or guardians, along with assent from the students.

The questionnaire was administered during school hours in spaces previously designated by the educational authorities, ensuring an appropriate environment for its completion. Students were organized into groups and received standardized instructions before responding. At all times, trained staff supervised the process to address questions and ensure its smooth execution. The estimated response time ranged from 25 to 30 min.

After data collection, the database underwent a validation and cleaning process to exclude incomplete or inconsistent records. For the present secondary data analysis, only participants who fully completed the sections of the questionnaire related to family dysfunction and ED risk were included, ensuring the quality of the analysis.

### 2.4. Variables

The dependent variable was ED risk, assessed using the SCOFF questionnaire (five dichotomous yes/no items; total score 0–5). A positive screen (indicative of ED risk) was defined as a score ≥ 2. The SCOFF is strictly a screening tool and does not provide a clinical diagnosis.

The exposure variable was family dysfunction. Family functioning was evaluated using the Family APGAR (five items scored 0–4; total score 0–20). Scores were categorized as: functional (18–20 points), mild dysfunction (14–17 points), moderate dysfunction (10–13 points), and severe dysfunction (0–9 points).

In addition, this study included various covariates of interest, grouped into sociodemographic, family, academic, behavioral, and mental health dimensions.

Within the sociodemographic characteristics, sex (male or female), type of educational institution (public or private), and place of residence (rural, urban, or peri-urban) were considered. Likewise, the number of family members was included, classified into three groups: 1–5, 6–10, and 11–15 members. Regarding family history and health background, the presence of a family history of mental illness (yes/no) was evaluated, as well as the adolescent’s nutritional status, determined through body mass index (BMI) and classified as underweight, normal weight, overweight, or obese.

Interpersonal relationships were analyzed through the frequency of contact with friends (infrequent, frequent, and very frequent). Academic performance was also assessed (poor, fair, good, and very good), as well as the history of failing any course during the school period (yes/no).

Regarding behavioral factors, the adolescent’s relationship status (in a relationship or single) was included, as well as alcohol consumption, measured at different frequencies (never, monthly or less, 2–4 times per month, 2–3 times per week, and 4 or more times per week), and cigarette use, categorized according to daily quantity (never, fewer than 10 cigarettes, 11–20, 21–30, and 31 or more cigarettes per day). It was also recorded whether the adolescent had ever sought mental health support (yes/no). The study also included variables related to technology use and digital exposure, such as frequency of social media use (never, low, moderate, high, and very high), and daily internet use (1–5 h, 6–10 h, and 11–15 h). With regard to COVID-19 history, it was considered whether the adolescent had family members who had been hospitalized due to the disease (yes/no).

Finally, several mental health and psychological well-being variables were included, such as low self-esteem (yes/no), level of resilience (low or high), presence of insomnia (absent, subclinical, moderate clinical, and severe clinical), and bullying victimization (yes/no). In addition, symptoms of depression, anxiety, and stress were assessed, each categorized as mild, moderate, severe, and extremely severe.

### 2.5. Instruments

#### 2.5.1. Risk for Eating Disorders (ED Risk)—SCOFF

For the screening of ED risk, the SCOFF questionnaire was used. It consists of five dichotomous items (yes/no) that correspond to five key clinical dimensions: self-induced vomiting (“Sick”), loss of control over eating (“Control”), significant weight loss (~6 kg in a short period; “One stone”), concern about being considered fat (“Fat”), and the perception that food dominates life (“Food”). The total score ranges from 0 to 5 and is classified as “no risk” (<2 points) or “at risk of ED” (≥2 points). In its original development, the SCOFF achieved a sensitivity of 100% and a specificity of 87.5% [29,30]. In Latin American contexts, it has been validated among school adolescents in Colombia (n = 241), where it showed a sensitivity of 81.9%, specificity of 78.7%, area under the ROC curve of 0.86, and a Cronbach’s alpha of 0.436, as well as moderate agreement with other diagnostic instruments [31].

#### 2.5.2. Family Functioning—Family APGAR

Family functioning was assessed using the Smilkstein Family APGAR scale. It consists of five items rated on a 4-point Likert scale (0 = never, 1 = almost never, 2 = sometimes, 3 = almost always, 4 = always). The total score ranges from 0 to 20, with higher scores indicating better functioning. Based on the total score, participants were classified as: functional (18–20 points), mild dysfunction (14–17 points), moderate dysfunction (10–13 points), and severe dysfunction (0–9 points) [32]. In Latin American contexts, it has been validated among school adolescents in Colombia (n = 1462), where internal reliability was adequate, with Cronbach’s alpha = 0.819 and McDonald’s omega = 0.820 [32].

Validated instruments were used to assess all study variables. Depressive, anxious, and stress symptoms were assessed using the Depression, Anxiety, and Stress Scale–21 items (DASS-21); insomnia symptoms were evaluated with the Insomnia Severity Index (ISI); self-esteem was measured using the Rosenberg Self-Esteem Scale; bullying experiences were assessed using the European Bullying Intervention Project Questionnaire (EBIPQ); and resilience was measured with the Connor-Davidson Resilience Scale–Short Form (CD-RISC-10). A detailed description of each instrument, including the number of items, scoring method, response scale, and psychometric properties in Latin American adolescent populations, is provided in Supplementary Material File S1 [33,34,35,36,37,38,39,40,41].

### 2.6. Statistical Analysis Plan

Data processing and analysis were performed using Stata version 18.0 (StataCorp LP, College Station, TX, USA). First, a descriptive analysis of the variables of interest was conducted. For categorical variables, absolute and relative frequencies were calculated, while for numerical variables, measures of central tendency and dispersion were determined after assessing normality. Subsequently, a bivariate analysis was carried out to explore the association between family dysfunction and the presence of ED risk. For this purpose, the chi-square test of independence was applied to categorical variables.

Prevalence ratios (PR) with 95% confidence intervals (95% CI) were estimated using Poisson regression models with robust variance to assess the magnitude of the association between variables. A significance level of 5% (*p* < 0.05) was established for all statistical tests performed.

In the multivariable analysis, Poisson regression models adjusted for clustering at the school level were used to control for intra-school correlation and avoid underestimation of variance. In addition, the association of interest (family dysfunction and ED risk) was adjusted for all secondary independent variables. Multicollinearity was assessed using the Variance Inflation Factor (VIF), considering values greater than 10 as indicative of high collinearity. All variables included in the final model had VIF values below this threshold, indicating acceptable levels of independence among predictors.

### 2.7. Ethical Aspects

The study was approved by the Research Ethics Committee of San Martín de Porres University, Lima, Peru, ensuring compliance with the principles of respect, beneficence, and justice in research involving human subjects. Informed consent was obtained from parents or guardians, and assent was obtained from adolescents, guaranteeing voluntary and confidential participation. Data management complied with international standards of privacy and confidentiality, in accordance with the Declaration of Helsinki. The questionnaires were anonymous, and results were analyzed in aggregate form, without individual references.

## 3. Results

### 3.1. Socio-Educational Characteristics of Adolescents

Among the 1219 adolescent participants, the mean age was 14.7 years, and the majority were male (55.4%), studied in public schools (65.1%), were in the second year of secondary education (22.9%), and resided in urban areas (82.9%). A total of 24.2% and 28.1% reported very frequent contact with their family and friends, respectively. In terms of behavioral factors, 13.0% reported drinking alcohol at least monthly, and 3.2% reported smoking fewer than 10 cigarettes per day. Regarding psychological well-being, 44.5% presented low self-esteem, 17.6% showed high levels of resilience, and 7.1% suffered from moderate insomnia. Additionally, 10.8%, 19.9%, and 2.9% reported extremely severe symptoms of depression, anxiety, and stress, respectively. With respect to family functioning, 10.0% and 29.8% of participants presented moderate and severe family dysfunction, respectively (Table 1).

### 3.2. Risk for Eating Disorders (ED Risk) in Adolescents

**Figure 1:**
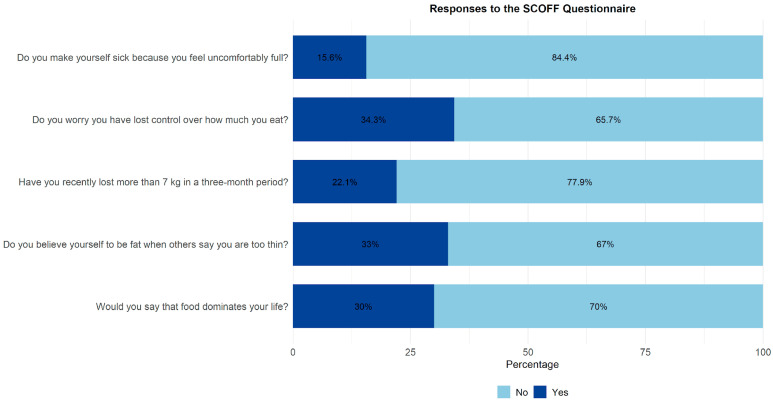
Responses to the SCOFF Questionnaire.

The screening positive for ED risk was 39.3% (95% CI: 36.54–42.10) in adolescents. A total of 34.3% reported being worried about having lost control over the amount of food they eat, 33% stated that they believe they are fat even though others say they are too thin, and 30% indicated that food dominates their life (Table 1 and Figure 1).

### 3.3. Family Dysfunction and Other Factors Associated with ED Risk, in Bivariate Analysis

The frequency of ED risk in adolescents with mild, moderate, and severe family dysfunction was 7.8%, 23.7%, and 3% higher, respectively, compared to adolescents with normal family functioning (Table 2). 

In addition, the frequency of ED risk was higher among female adolescents (55% vs. 26.7%; *p* < 0.001), those attending public schools (41.8% vs. 34.6%; *p* = 0.014), those with a family history of mental illness (59.6% vs. 35.8%; *p* < 0.001), those consuming alcohol 4 or more times per week (61.5% vs. 35.8%; *p* < 0.001), those who sought mental health support during the pandemic (52.4% vs. 35.9%; *p* < 0.001), those with low self-esteem (49.9% vs. 30.8%; *p* < 0.001), those with low resilience (41.3% vs. 29.8%; *p* = 0.002), those with severe insomnia (82.1% vs. 27.3%; *p* < 0.001), those with extremely severe depressive symptoms (73.5% vs. 17.8%; *p* < 0.001), those with extremely severe anxiety symptoms (69.4% vs. 18.9%; *p* < 0.001), and those with extremely severe stress symptoms (91.4% vs. 25.7%; *p* < 0.001) (Table 2). 

### 3.4. Family Dysfunction and Other Factors Associated with ED Risk, in Simple and Multiple Regression Analysis

In the simple regression model, adolescents with moderate family dysfunction had a 69% higher ED risk (PR: 1.69; 95% CI: 1.49–1.92) (Table 3). 

In the multivariable model, this association remained significant, with adolescents showing 11% higher ED risk (PR: 1.11; 95% CI: 1.01–1.22) (Table 4).

Other factors were also associated with higher ED risk. Obesity was associated with a 17% higher ED risk (PR: 1.17; 95% CI: 1.04–1.30). Having a family member with a mental illness was associated with an 18% higher ED risk (PR: 1.18; 95% CI: 1.01–1.39). Failing a course during school years was associated with an 18% higher ED risk (PR: 1.18; 95% CI: 1.06–1.31). Alcohol consumption four or more times per week was associated with a 75% higher ED risk (PR: 1.75; 95% CI: 1.29–2.37). Having a family member hospitalized for COVID-19 was associated with a 14% higher ED risk (PR: 1.14; 95% CI: 1.03–1.26). Living with 11 to 15 people at home was associated with a 24% higher ED risk (PR: 1.24; 95% CI: 1.10–1.39). Moderate insomnia was associated with a 61% higher ED risk (PR: 1.61; 95% CI: 1.25–2.07). Finally, having mild, severe, and extremely severe depressive symptoms was associated with 68% (PR: 1.68; 95% CI: 1.38–2.05), 80% (PR: 1.80; 95% CI: 1.27–2.55), and 68% (PR: 1.68; 95% CI: 1.10–2.54) higher ED risk, respectively (Table 4).

Overall, the results show a high burden of ED risk concentrated among adolescents exposed to family dysfunction and multiple psychosocial stressors, while social resources (such as frequent peer contact) and individual assets (such as resilience) aligned with lower ED risk.

## 4. Discussion

### 4.1. Association Between Family Dysfunction and ED Risk

Adolescents with moderate family dysfunction had a higher ED risk. This is consistent with a study conducted in Colombia, severe family dysfunction was associated with a higher ED risk (OR = 2.3) [22]. Among Venezuelan adolescents, family dysfunction was linked to a greater risk of binge-type ED symptoms (PR = 2.76) [24]. Similarly, Mexican adolescents demonstrated higher screening-based ED risk among females [42], and a Peruvian study in pre-university students found that family dysfunction increased the likelihood of screening positive for ED risk (OR = 2.46) [25].

This may be associated with role of the family and its determining influence in preventing the development of such disorders, as it represents a cornerstone in the formation of the individual [43]. This is further supported by findings from a study in adolescents where family connectedness was associated with a protective effect against ED risk [44]. One point to consider is that dysfunctional families often exhibit poor communication among members, generating a chaotic environment in which adolescents may be more predisposed to avoiding both internal and external problems. As a result, they may be less likely to seek help and may engage in behaviors that pose health risks, such as screening positive for ED risk [45,46].

In our adjusted model, moderate family dysfunction remained significantly associated with a higher ED risk, whereas severe dysfunction did not. This pattern does not imply a protective effect of severe dysfunction; instead, it is most plausibly explained by statistical attenuation after adjustment. In the crude model, severe dysfunction showed the expected positive association with ED risk, but this effect diminished once multiple psychosocial and behavioral covariates were included. This attenuation is consistent with confounding-induced suppression, a phenomenon where factors highly correlated with both the exposure (severe dysfunction) and the outcome (ED risk)—such as depressive symptoms, insomnia, previous mental illness, low resilience, and COVID-19–related family stressors—reallocate part of the variance previously attributed to the main predictor. As a result, the adjusted estimate loses magnitude and statistical significance. Therefore, the non-significant association observed for severe dysfunction should be interpreted cautiously and understood as a modeling artifact rather than a deviation from the theoretically expected gradient, which longitudinal studies would better elucidate.

### 4.2. Risk for Eating Disorders (ED Risk)

We found that nearly 4 out of 10 adolescents screened positive for ED risk (39.2%). This level of ED risk is much higher than what has been reported in pre-pandemic studies: in Chilean adolescents, 8.3% presented ED risk [8], and in Venezuelan adolescents, 7.8% presented ED risk for binge-type ED risk symptoms based on a screening tool [24]; and a 2015 Peruvian study in adolescent girls reported a 20.2% ED risk [47]. In the context of the COVID-19 pandemic, our PubMed-based review found no population-level epidemiologic studies on adolescent ED risk. Available evidence comprised a letter to the editor [48] and an official newspaper report [49], both suggesting apparent increases in diagnoses among individuals aged ≥11 years. This discrepancy may be associated with the timing of the studies, as the aforementioned research was conducted prior to the psychosocial changes brought about by the COVID-19 pandemic.

Adolescents represented a high-risk group during the pandemic, and the high ED risk may be associated with compensatory behaviors in response to the impact of uncertainty, frustration, boredom, misinformation, and possible economic difficulties, among other stressors experienced in this context. These conditions may be associated with increased emotional stress, which may, in turn, be associated with changes in eating behavior and weight control [50].

Moreover, during this period there was a marked increase in the use of social media, a variable not assessed in this study. However, previous research has reported its association with ED risk, pointing to sociocultural pressures favoring thinness and the stereotype of the “perfect body” exerted on adolescents, which may have contributed to the development of unhealthy eating patterns [51,52].

Additionally, school closures and the implementation of distance learning deprived many adolescents of access to mental health services, social support, and extracurricular activities; the lack of social interaction and reduced physical activity may have been associated with changes in eating behaviors and increased ED risk [50,53].

### 4.3. Other Factors Associated with ED Risk

Being male was associated with a lower ED risk, a result consistent with findings from a study in Colombian adolescents, where being female was associated with a higher likelihood of screening positive for ED risk (OR = 4.41) [54]. This may be explained by beauty stereotypes and the social pressure generated by today’s media, which may be associated with greater vulnerability among girls due to stronger sociocultural emphasis on body image [51].

Living in peri-urban areas was associated with a lower ED risk. This finding should be interpreted cautiously, as it may partly reflect differences in lifestyle, food access, or exposure to sociocultural pressures between peri-urban and urban adolescents. Adolescents living in peri-urban settings may have reduced exposure to media-driven body ideals or distinct dietary and social environments, which may be associated with lower levels of ED risk vulnerability [54,55,56]. However, this interpretation remains speculative and warrants further research using more detailed measures of socioeconomic context and media exposure.

Obesity was associated with a higher ED risk. This aligns with a Venezuelan study in which adolescents with generalized obesity had a greater risk of binge-type ED behaviors (PR = 3.20) [24], and with findings in Spanish adolescents showing higher odds of ED risk among those with overweight (OR = 4.91) [56]. This may be associated with body dissatisfaction experienced by obese adolescents due to stigmatization from those around them, which may be associated with engagement in compensatory behaviors such as vomiting, purging, or laxative use [57,58].

Having a family member with a mental health problem was associated with a higher ED risk. Prior reviews note that parental psychopathology is strongly linked to ED vulnerability and treatment outcomes among adolescents [59]. The explanation for this association may be that adolescents with a family history of mental health problems are more frequently exposed to stressful situations and family conflicts, which increases their risk of developing mental health problems such as ED risk. Additionally, adolescents may learn unhealthy behaviors from their relatives, leading to an increased likelihood of ED risk [59]. This association may reflect greater exposure to stressful family environments, which may be associated with increased emotional distress, as well as social learning mechanisms through which adolescents may adopt maladaptive behaviors observed in relatives [55,60].

Frequent contact with friends was associated with a lower ED risk. This contrasts with findings among U.S. female adolescents, where greater peer interaction increased ED risk [44]. Such discrepancies may reflect sociocultural differences in the role of peer relationships, since in developing contexts, friendship networks may provide emotional support and buffer stress rather than reinforce appearance-related pressures [55]. Social connectedness has been shown to reduce stress and improve appetite regulation, which may partly account for its protective effect [61,62,63].

Failing a course was associated with a higher ED risk. This is consistent with evidence that school connectedness and academic performance are inversely related to ED risk (OR = 0.69) [44]. This association may be explained by the fact that academic difficulties may be associated with increased stress, anxiety, and low self-esteem, which can manifest as maladaptive ED patterns [63].

Frequent alcohol consumption was associated with a higher ED risk. This finding aligns with international evidence showing that substance use and ED often co-occur [44,64,65]. Alcohol use may disrupt impulse control and appetite regulation, which may be associated with ED risk [63,66]. These results underscore the importance of coexisting risky behaviors when designing adolescent mental health interventions.

Having a family member hospitalized with COVID-19 was associated with a higher ED risk. No studies have been identified that specifically evaluated this association. However, it is known that the consequences of COVID-19 affected not only the physical but also the psychological sphere; in fact, cases of ED have been reported in which family bereavement was identified as a trigger for the exacerbation of the disorder [48,67]. Having a family member hospitalized due to COVID-19 may have been associated with increased stress and anxiety symptoms within the family, potentially shaping the mental health of adolescents—particularly their eating behavior—due to concerns about their relatives’ health, coupled with limited access to medical care and financial difficulties caused by the pandemic [68].

Living with 11–15 people in the household was associated with a higher ED risk. This is consistent with findings from a study in China, where living in overcrowded conditions was found to increase the likelihood of experiencing mood disturbances (OR = 1.12), thereby negatively affecting residents’ mental health [69]. This may be associated with economic and psychological factors within the family: in households with a large number of members, there may be greater competition for resources, which could contribute to increased stress and anxiety among adolescents, and in turn may be associated with alterations in eating behavior [63]. In addition, households with a high number of residents may face food insecurity and inequities in access to health care services, both of which may influence ED risk [70].

Adolescents with a high level of resilience were associated with a lower ED risk. This finding is supported by results from a longitudinal study in Spanish patients with a mean age of 30 years, where resilience predicted a reduction in ED symptoms [71], and also by a qualitative study in Spanish women diagnosed with ED, which reported that resilience preceded the recovery experience [72]. This may be associated with the fact that individuals with high levels of resilience are better able to adapt to adversity, have greater problem-solving skills, and make effective decisions, which may support a good level of emotional well-being. This, in turn, may help them maintain regular eating patterns, avoid disordered eating behaviors, and therefore present a lower ED risk [44].

Having moderate clinical insomnia was associated with a higher ED risk. This is consistent with findings from a study in Sweden, conducted among women aged 18 to 23 years, which reported an association between difficulty maintaining sleep and the perception of non-restorative sleep with the presence of ED risk [73]. This may be associated with the self-focused concern experienced by these adolescents, generating a state of “hyperarousal” that prevents them from achieving restorative sleep. Additionally, sleep and eating behaviors are regulated by similar neurobiological systems, including appetite and satiety regulation, the reward system, and the stress system [74]. Sleep disorders such as insomnia may disrupt the neurobiological regulation of these systems, thereby being associated with a higher probability of disordered eating behaviors [75].

Depressive and anxiety symptoms were associated with a higher ED risk. This finding is consistent with studies in Venezuela, Colombia, and international surveys reporting a strong overlap between affective disorders and disordered eating behaviors [24,54,64]. Both conditions share mechanisms of emotional dysregulation and stress reactivity that may alter appetite and reward pathways, potentially contributing to maladaptive coping through food restriction or overeating [55,62,63,74,76]. These results highlight the need for integrated screening and interventions addressing mood symptoms within ED prevention programs for adolescents.

### 4.4. Relevance of Findings in Mental Health

The findings of this study have significant implications for the field of mental health, particularly regarding the understanding and management of ED risk in Peruvian adolescents. The association between family dysfunction and a higher ED risk highlights the crucial role of the family environment in the development and progression of this condition. Additionally, this research makes it possible to assess multiple factors associated with ED risk—including personal, family, socioeconomic, and pandemic-related circumstances—that may serve as a reference for future situations generating high stress in this population, which is inherently vulnerable to adverse events, as previously discussed.

These findings emphasize the importance of early detection and intervention in cases of family dysfunction. Health professionals should be aware of the potential influence of family dysfunction on adolescent mental health and incorporate this knowledge into their assessment and treatment strategies. Interventions aimed at improving family functioning and communication skills may help mitigate the ED risk and enhance overall mental well-being in adolescents, as well as in the caregivers who support them throughout the recovery process [77,78]. By identifying and addressing family dysfunction early, health professionals can contribute to the prevention and reduction in ED risk, ultimately leading to a better quality of life for adolescents during their school years.

### 4.5. Limitations and Strengths

Among the limitations of this study, the most relevant is its cross-sectional nature, which prevents establishing a causal relationship between ED risk and the variables studied. In addition, there is a risk of selection bias, as our results cannot be generalized to the entire Peruvian adolescent population; nevertheless, participation was successfully recruited from five secondary schools in the Lambayeque region. There is also the possibility of information bias, since certain potential confounding variables for ED risk, which would have been of interest, could not be measured, such as concern with appearance [44], body dissatisfaction [62], media influence [51], socioeconomic status [54], and physical condition [56]. Additionally, the use of the SCOFF questionnaire represents an important methodological limitation. Although it is a brief and widely validated screening tool for detecting ED risk, it does not allow clinical diagnosis, and its sensitivity and specificity may vary according to population characteristics and cultural context. Recent evidence suggests that the SCOFF may overestimate risk when used in non-clinical adolescent samples and that cross-cultural adaptation is essential to improve accuracy [79]. Therefore, our results should be interpreted as indicative of risk rather than prevalence of ED. Although multicollinearity diagnostics indicated acceptable levels (VIF < 10), the inclusion of correlated psychological constructs—such as depression, anxiety, stress, and insomnia may have contributed to attenuated some effect estimates due to partial overlap among these variables. Additionally, the exclusion of 217 incomplete cases may have introduced minor selection bias; however, these exclusions represented less than 10% of the total sample and were not clustered in any specific subgroup.

Beyond the SCOFF, the instruments employed have inherent measurement constraints. The Family APGAR provides a brief, subjective assessment of perceived family functioning, which may not fully capture deeper structural or interactional dynamics. However, its simplicity and validated Spanish version support its use in large-scale adolescent studies in Latin America [80]. Conversely, the SCOFF’s strength lies in its rapid detection of risk, yet it may produce false positives if applied outside clinical settings. Together, these instruments offer practical, psychometrically supported tools for population-level screening, while underscoring the need for complementary diagnostic evaluations in future research [30].

Despite these limitations, this study has several strengths. These include the use of validated instruments in populations similar to the one studied [45,56]; the sample size provided statistical power greater than 80%, allowing for more reliable extrapolation of the results obtained; and in-person data collection, which enabled clarification of participants’ doubts while completing the questionnaire. Finally, to the best of our knowledge, this is one of the first studies to evaluate whether family dysfunction is associated with ED risk in adolescents during the COVID-19 pandemic in Latin America.

Beyond methodological considerations, these findings should be interpreted within the sociocultural context of Peruvian adolescents, where family cohesion, peer relationships, and school environments strongly influence emotional and behavioral development [16,20,81]. Although the results primarily reflect local realities, they may also be extrapolated to other Latin American populations that share similar family and educational dynamics [8,9,21]. From a preventive perspective, the results highlight the need for integrated mental health and health promotion efforts, particularly school and family-based programs that strengthen communication, resilience, and social support networks [28,46,78]. Collaborative approaches involving families, peers, and schools may play a critical role in early detection and intervention to reduce the ED risk and improve adolescent well-being [30,31,79].

## 5. Conclusions

Moderate family dysfunction was associated with a higher ED risk, highlighting the pivotal role of family environment in shaping adolescents’ emotional regulation and coping behaviors. Dysfunctional family dynamics—marked by poor communication, conflict, and limited emotional support—may be associated with a greater likelihood of adolescents adopting maladaptive ED patterns in response to stress or low self-esteem. The observed high ED risk during the post-pandemic period may reflect persistent psychosocial disruptions, possibly linked to reduced access to mental health services, peer interaction, and structured extracurricular activities.

Conversely, protective associations were identified for male sex, frequent peer contact, and higher resilience, emphasizing the relevance of social support and adaptive coping in mitigating ED risk. In contrast, obesity, family mental health history, academic failure, alcohol consumption, family hospitalization due to COVID-19, overcrowding, insomnia, depression, and anxiety were associated with greater vulnerability.

Overall, these findings reinforce the need for school-based preventive programs that integrate family functioning assessments, resilience training, and mental health promotion. As the SCOFF is a screening instrument rather than a diagnostic tool, these results represent population-level risk estimates rather than confirmed clinical cases, underscoring the importance of longitudinal studies to further elucidate potential pathways underlying these associations.

Additionally, the results should be interpreted within the cultural context of Peruvian adolescents, where family cohesion, peer networks, and school environments are central to psychosocial development. Although the findings primarily reflect local conditions, they may be extrapolated to other Latin American settings with similar family and educational dynamics. These insights underscore the importance of culturally adapted, multi-level prevention strategies that involve families, peers, and schools to strengthen adolescent mental health and reduce vulnerability to ED risk.

## Figures and Tables

**Table 1:** Characteristics of adolescents from Chiclayo, Peru.

Characteristics		N (%)
Age		14.66 ± 1.39
Sex		
	Male	544 (44.6)
	Female	675 (55.4)
Type of institution		
	Public	794 (65.1)
	Particular	425 (34.9)
Place of residence		
	Rural	176 (14.4)
	Urban	1011 (82.9)
	Marginal urban	32 (2.6)
Number of family members (categorized)		
	1 to 5	724 (59.4)
	6 to 10	447 (36.7)
	11 to 15	48 (3.9)
Family history of mental illness		
	No	1041 (85.4)
	Yes	178 (14.6)
BMI (categorized)		
	Underweight	253 (20.8)
	Normal weight	765 (62.8)
	Overweight	160 (13.1)
	Obesity	41 (3.4)
Contact with friends		
	Infrequent	300 (24.6)
	Frequent	577 (47.3)
	Very frequent	342 (28.1)
Academic performance		
	Poor	28 (2.3)
	Fair	44 (3.6)
	Good	486 (39.9)
	Very good	507 (41.6)
	Very frequent	154 (12.6)
Failed any school course		
	No	667 (54.7)
	Yes	552 (45.3)
In a relationship		
	No	451 (37.0)
	Yes	768 (63.0)
Alcohol consumption		
	Never	945 (77.5)
	Monthly or less	158 (13.0)
	2 to 4 times a month	78 (6.4)
	2 to 3 times a week	25 (2.1)
	4 or more times a week	13 (1.1)
Cigarette consumption		
	Never	1145 (93.9)
	Less than 10 cigarettes/day	39 (3.2)
	11 to 20 cigarettes/day	21 (1.7)
	21 to 30 cigarettes/day	5 (0.4)
	31 or more cigarettes/day	9 (0.7)
Sought mental health support		
	No	971 (80.0)
	Yes	248 (20.3)
Frequency of social media use		
	Never	105 (8.6)
	Rarely	271 (22.2)
	Moderate	336 (27.6)
	Quite often	394 (32.3)
	Extreme	113 (9.3)
Daily internet use time (in hours)		
	1 to 5	752 (61.7)
	6 to 10	276 (22.6)
	11 to 15	191 (15.7)
Family member hospitalized due to COVID-19		
	No	598 (49.1)
	Yes	621 (50.9)
Low self-esteem		
	No	676 (55.5)
	Yes	543 (44.5)
Resilience		
	Low	1004 (82.4)
	High	215 (17.6)
Insomnia		
	No	744 (61.0)
	Subclinical	361 (29.6)
	Moderate clinical	86 (7.1)
	Severe clinical	28 (2.3)
Victim of bullying		
	No	784 (64.3)
	Yes	435 (35.7)
Depression		
	No	472 (38.7)
	Mild	175 (14.4)
	Moderate	295 (24.2)
	Severe	145 (11.9)
	Extremely severe	132 (10.8)
Anxiety		
	No	439 (36.0)
	Mild	106 (8.7)
	Moderate	292 (24.0)
	Severe	140 (11.5)
	Extremely severe	242 (19.9)
Stress		
	No	716 (58.7)
	Mild	187 (15.3)
	Moderate	188 (15.4)
	Severe	93 (7.6)
	Extremely severe	35 (2.9)
Family dysfunction		
	Functional	493 (40.4)
	Mild	241 (19.8)
	Moderate	122 (10.0)
	Severe	363 (29.8)
ED risk		
	No	740 (60.7)
	Yes	479 (39.3)
Mean ± standard deviation		

**Table 2:** Factors associated with ED risk, in bivariate analysis.

Variables		Risk for Eating Disorders (ED Risk)		*p* *
		No (n = 740)	Yes (n = 479)	
		n (%)	n (%)	
Sex				<0.001
	Male	245 (45.0)	299 (55.0)	
	Female	495 (73.3)	180 (26.7)	
Type of institution				0.014
	Public	462 (58.2)	332 (41.8)	
	Particular	278 (65.4)	147 (34.6)	
Place of residence				0.119
	Rural	99 (56.3)	77 (43.8)	
	Urban	617 (61.0)	394 (39.0)	
	Marginal urban	24 (75.0)	8 (25.0)	
Number of family members (categorized)				0.289
	1 to 5	445 (61.5)	279 (38.5)	
	6 to 10	271 (60.6)	176 (39.4)	
	11 to 15	24 (50.0)	24 (50.0)	
Family history of mental illness				<0.001
	No	668 (64.2)	373 (35.8)	
	Yes	72 (40.5)	106 (59.6)	
BMI (categorized)				0.226
	Underweight	164 (64.8)	89 (35.2)	
	Normal weight	465 (60.8)	300 (39.2)	
	Overweight	88 (55.0)	72 (45.0)	
	Obesity	23 (56.1)	18 (43.9)	
Contact with friends				<0.001
	Infrequent	173 (57.7)	127 (42.3)	
	Frequent	389 (67.4)	188 (32.6)	
	Very frequent	178 (52.1)	164 (48.0)	
Academic performance				0.034
	Poor	16 (57.1)	12 (42.9)	
	Fair	23 (52.3)	21 (47.7)	
	Good	276 (56.8)	210 (43.2)	
	Very good	318 (62.7)	189 (37.3)	
	Very frequent	107 (69.5)	47 (30.5)	
Failed any school course				<0.001
	No	437 (65.5)	230 (34.5)	
	Yes	303 (54.9)	249 (45.1)	
In a relationship				<0.001
	No	303 (67.2)	148 (32.8)	
	Yes	437 (56.9)	331 (43.1)	
Alcohol consumption				<0.001
	Never	607 (64.2)	338 (35.8)	
	Monthly or less	83 (52.5)	75 (47.5)	
	2 to 4 times a month	32 (41.0)	46 (59.0)	
	2 to 3 times a week	13 (52.0)	12 (48.0)	
	4 or more times a week	5 (38.5)	8 (61.5)	
Cigarette consumption				0.136
	Never	704 (61.5)	441 (38.5)	
	Less than 10 cigarettes/day	17 (43.6)	22 (56.4)	
	11 to 20 cigarettes/day	13 (61.9)	8 (38.1)	
	21 to 30 cigarettes/day	2 (40.0)	3 (60.0)	
	31 or more cigarettes/day	4 (44.4)	5 (55.6)	
Sought mental health support				<0.001
	No	622 (64.1)	349 (35.9)	
	Yes	118 (47.6)	130 (52.4)	
Frequency of social media use				0.001
	Never	73 (69.5)	32 (30.5)	
	Rarely	171 (63.1)	100 (36.9)	
	Moderate	218 (64.9)	118 (35.1)	
	Quite often	227 (57.6)	167 (42.4)	
	Extreme	51 (45.1)	62 (54.9)	
Daily internet use time (in hours)				0.037
	1 to 5	477 (63.4)	275 (36.6)	
	6 to 10	159 (57.6)	117 (42.4)	
	11 to 15	104 (54.5)	87 (45.6)	
Family member hospitalized due to COVID-19				0.010
	No	385 (64.4)	213 (35.6)	
	Yes	355 (57.2)	266 (42.8)	
Low self-esteem				<0.001
	No	468 (69.2)	208 (30.8)	
	Yes	272 (50.1)	271 (49.9)	
Resilience				0.002
	Low	589 (58.7)	415 (41.3)	
	High	151 (70.2)	64 (29.8)	
Insomnia				<0.001
	No	541 (72.7)	203 (27.3)	
	Subclinical	177 (49.0)	184 (51.0)	
	Moderate clinical	17 (19.8)	69 (80.2)	
	Severe clinical	5 (17.9)	23 (82.1)	
Victim of bullying				
	No	488 (62.2)	296 (37.8)	0.140
	Yes	252 (57.9)	183 (42.1)	
Depression				<0.001
	No	388 (82.2)	84 (17.8)	
	Mild	104 (59.4)	71 (40.6)	
	Moderate	165 (55.9)	130 (44.1)	
	Severe	48 (33.1)	97 (66.9)	
	Extremely severe	35 (26.5)	97 (73.5)	
Anxiety				<0.001
	No	356 (81.1)	83 (18.9)	
	Mild	80 (75.5)	26 (24.5)	
	Moderate	161 (55.1)	131 (44.9)	
	Severe	69 (49.3)	71 (50.7)	
	Extremely severe	74 (30.6)	168 (69.4)	
Stress				<0.001
	No	532 (74.3)	184 (25.7)	
	Mild	97 (51.9)	90 (48.1)	
	Moderate	78 (41.5)	110 (58.5)	
	Severe	30 (32.3)	63 (67.7)	
	Extremely severe	3 (8.6)	32 (91.4)	
Family dysfunction				<0.001
	Functional	323 (65.5)	170 (34.5)	
	Mild	139 (57.7)	102 (42.3)	
	Moderate	51 (41.8)	71 (58.2)	
	Severe	227 (62.5)	136 (37.5)	

* *p*-value calculated using the Chi-square test of independence.

**Table 3:** Association between family dysfunction and other factors related to ED risk in adolescents: simple regression analysis.

Variables		Risk for Eating Disorders (ED Risk)		
		Simple Regression		
		PR	CI 95%	*p* *
Sex				
	Male	Ref.		
	Female	0.48	0.43–0.55	<0.001
Type of institution				
	Public	Ref.		
	Particular	0.82	0.80–0.84	<0.001
Place of residence				
	Rural	Ref.		
	Urban	0.91	0.72–1.14	0.399
	Marginal urban	0.58	0.45–0.75	<0.001
Number of family members (categorized)				
	1 to 5	Ref.		
	6 to 10	1.03	0.91–1.16	0.634
	11 to 15	1.28	0.82–1.98	0.276
Family history of mental illness				
	No	Ref.		
	Yes	1.67	1.41–1.97	<0.001
BMI (categorized)				
	Underweight	Ref.		
	Normal weight	1.11	0.99–1.26	0.084
	Overweight	1.28	0.96–1.70	0.087
	Obesity	1.25	0.99–1.58	0.066
Contact with friends				
	Infrequent	Ref.		
	Frequent	0.76	0.69–0.85	<0.001
	Very frequent	1.11	0.92–1.33	0.279
Academic performance				
	Poor	Ref.		
	Fair	1.14	0.89–1.47	0.307
	Good	1.00	0.86–1.17	0.986
	Very good	0.87	0.71–1.07	0.184
	Very frequent	0.71	0.52–0.98	0.037
Failed any school course				
	No	Ref.		
	Yes	1.31	1.10–1.55	0.002
In a relationship				
	No	Ref.		
	Yes	1.34	1.10–1.62	0.004
Alcohol consumption				
	Never	Ref.		
	Monthly or less	1.32	1.15–1.52	<0.001
	2 to 4 times a month	1.67	1.45–1.92	<0.001
	2 to 3 times a week	1.35	0.98–1.84	0.063
	4 or more times a week	1.73	1.31–2.28	<0.001
Cigarette consumption				
	Never	Ref.		
	Less than 10 cigarettes/day	1.47	1.05–2.05	0.025
	11 to 20 cigarettes/day	0.99	0.89–1.10	0.868
	21 to 30 cigarettes/day	1.56	1.14–2.14	0.006
	31 or more cigarettes/day	1.45	1.29–1.62	<0.001
Sought mental health support				
	No	Ref.		
	Yes	1.48	1.34–1.64	<0.001
Frequency of social media use				
	Never	Ref.		
	Rarely	1.23	0.93–1.61	0.148
	Moderate	1.16	0.92–1.45	0.208
	Quite often	1.40	1.19–1.65	<0.001
	Extreme	1.83	1.28–2.62	0.001
Daily internet use time (in hours)				
	1 to 5	Ref.		
	6 to 10	1.17	1.01–1.36	0.037
	11 to 15	1.25	1.08–1.44	0.002
Family member hospitalized due to COVID-19				
	No	Ref.		
	Yes	1.21	1.03–1.42	0.021
Low self-esteem				
	No	Ref.		
	Yes	1.61	1.18–2.21	0.003
Resilience				
	Low	Ref.		
	High	0.71	0.59–0.86	0.001
Insomnia				
	No	Ref.		
	Subclinical	1.87	1.54–2.26	<0.001
	Moderate clinical	2.89	2.37–3.53	<0.001
	Severe clinical	3.04	2.08–4.44	<0.001
Victim of bullying				
	No	Ref.		
	Yes	1.11	0.96–1.29	0.146
Depression				
	No	Ref.		
	Mild	2.30	1.98–2.67	<0.001
	Moderate	2.50	1.93–3.24	<0.001
	Severe	3.77	3.24–4.39	<0.001
	Extremely severe	4.08	3.39–4.92	<0.001
Anxiety				
	No	Ref.		
	Mild	1.22	0.92–1.63	0.172
	Moderate	2.28	2.06–2.52	<0.001
	Severe	2.62	2.29–2.99	<0.001
	Extremely severe	3.52	3.40–3.65	<0.001
Stress				
	No	Ref.		
	Mild	1.86	1.71–2.01	<0.001
	Moderate	2.25	1.89–2.69	<0.001
	Severe	2.58	2.10–3.18	<0.001
	Extremely severe	3.54	2.84–4.42	<0.001
Family dysfunction				
	Functional	Ref.		
	Mild	1.23	0.97–1.56	0.095
	Moderate	1.69	1.49–1.92	<0.001
	Severe	1.09	0.75–1.57	0.660

* *p*-values obtained using G*p-values obtained generalized Linear Models (GLM), Poisson family, log link function, robust variance, with school as the cluster.

**Table 4:** Association between family dysfunction and other factors related to ED risk in adolescents: multiple regression analysis.

Variables		Risk for Eating Disorders (ED Risk)		
		Multiple Regression *		
		PR	CI 95%	*p* **
Sex				
	Male	Ref.		
	Female	0.65	0.52–0.80	<0.001
Type of institution				
	Public	Ref.		
	Particular	0.95	0.87–1.04	0.267
Place of residence				
	Rural	Ref.		
	Urban	0.89	0.78–1.01	0.073
	Marginal urban	0.58	0.44–0.75	<0.001
Number of family members (categorized)				
	1 to 5	Ref.		
	6 to 10	0.99	0.82–1.20	0.939
	11 to 15	1.24	1.10–1.39	<0.001
Family history of mental illness				
	No	Ref.		
	Yes	1.18	1.01–1.39	0.042
BMI (categorized)				
	Underweight	Ref.		
	Normal weight	1.06	0.90–1.25	0.453
	Overweight	1.24	0.99–1.54	0.057
	Obesity	1.17	1.04–1.30	0.007
Contact with friends				
	Infrequent	Ref.		
	Frequent	0.85	0.79–0.93	<0.001
	Very frequent	1.12	0.98–1.29	0.107
Academic performance				
	Poor	Ref.		
	Fair	0.91	0.63–1.33	0.628
	Good	0.94	0.86–1.03	0.167
	Very good	1.05	0.82–1.33	0.712
	Very frequent	1.03	0.86–1.24	0.735
Failed any school course				
	No	Ref.		
	Yes	1.18	1.06–1.31	0.003
In a relationship				
	No	Ref.		
	Yes	1.19	0.98–1.43	0.075
Alcohol consumption				
	Never	Ref.		
	Monthly or less	1.04	0.73–1.47	0.839
	2 to 4 times a month	1.26	0.94–1.67	0.119
	2 to 3 times a week	1.26	0.84–1.87	0.274
	4 or more times a week	1.75	1.29–2.37	<0.001
Cigarette consumption				
	Never	Ref.		
	Less than 10 cigarettes/day	1.09	0.82–1.44	0.567
	11 to 20 cigarettes/day	0.83	0.60–1.14	0.241
	21 to 30 cigarettes/day	1.16	0.76–1.77	0.494
	31 or more cigarettes/day	1.02	0.70–1.48	0.931
Sought mental health support				
	No	Ref.		
	Yes	1.03	0.89–1.18	0.696
Frequency of social media use				
	Never	Ref.		
	Rarely	0.90	0.65–1.25	0.541
	Moderate	0.86	0.63–1.17	0.324
	Quite often	0.85	0.73–0.99	0.049
	Extreme	0.86	0.70–1.05	0.139
Daily internet use time (in hours)				
	1 to 5	Ref.		
	6 to 10	0.99	0.89–1.13	0.985
	11 to 15	1.01	0.88–1.16	0.908
Family member hospitalized due to COVID-19				
	No	Ref.		
	Yes	1.14	1.03–1.26	0.009
Low self-esteem				
	No	Ref.		
	Yes	1.12	0.95–1.32	0.180
Resilience				
	Low	Ref.		
	High	0.85	0.77–0.93	<0.001
Insomnia				
	No	Ref.		
	Subclinical	1.28	1.04–1.58	0.021
	Moderate clinical	1.61	1.25–2.07	<0.001
	Severe clinical	1.55	1.00–2.40	0.052
Victim of bullying				
	No	Ref.		
	Yes	1.07	0.97–1.17	0.178
Depression				
	No	Ref.		
	Mild	1.68	1.38–2.05	<0.001
	Moderate	1.48	0.99–2.21	0.056
	Severe	1.80	1.27–2.55	0.001
	Extremely severe	1.68	1.10–2.54	0.015
Anxiety				
	No	Ref.		
	Mild	0.96	0.64–1.43	0.834
	Moderate	1.44	1.13–1.84	0.003
	Severe	1.19	0.87–1.64	0.281
	Extremely severe	1.38	1.07–1.79	0.013
Stress				
	No	Ref.		
	Mild	1.11	1.01–1.23	0.030
	Moderate	1.20	1.05–1.38	0.008
	Severe	1.04	0.85–1.28	0.700
	Extremely severe	1.09	0.88–1.35	0.438
Family dysfunction				
	Functional	Ref.		
	Mild	1.00	0.94–1.06	0.992
	Moderate	1.11	1.01–1.22	0.039
	Severe	0.88	0.76–1.01	0.069

* Adjusted for covariates o*Adjusted for covariates of interest. ** *p*-values obtained using Generalized Linear Models (GLM), Poisson family, log link function, robust variance, with school as the cluster.

## Data Availability

Due to confidentiality and ethical restrictions, the datasets generated and/or analyzed during the current study are not publicly available. However, de-identified data may be made available to qualified researchers upon reasonable request from the corresponding author at vgarrido@uss.edu.pe.

## Supplementary Materials

The following supporting information can be downloaded at: https://www.mdpi.com/article/10.3390/jcm15051726/s1. Supplementary Material File S1. Description_of_measurement_instruments.pdf: Instrument Section Revision.

## Abbreviations

The following abbreviations are used in this manuscript:

ED risk	Risk for eating disorders
ED	Eating disorders
EBIPQ	European Bullying Intervention Project Questionnaire
ISI	Insomnia Severity Index
WHO	World Health Organization

## References

[1] Lyke J., Matsen J. (2013). Family functioning and risk factors for disordered eating. Eat. Behav..

[2] Ciao A.C., Accurso E.C., Fitzsimmons-Craft E.E., Lock J., Le Grange D. (2015). Family functioning in two treatments for adolescent anorexia nervosa. Int. J. Eat. Disord..

[3] Mohammadi M.R., Mostafavi S., Hooshyari Z., Khaleghi A., Ahmadi N., Molavi P., Kian A.A., Safavi P., Delpisheh A., Talepasand S. (2020). Prevalence, correlates and comorbidities of feeding and eating disorders in a nationally representative sample of Iranian children and adolescents. Int. J. Eat. Disord..

[4] Hammerle F., Huss M., Ernst V., Bürger A. (2016). Thinking dimensional: Prevalence of DSM-5 early adolescent full syndrome, partial and subthreshold eating disorders in a cross-sectional survey in German schools. BMJ Open.

[5] Sparti C., Santomauro D., Cruwys T., Burgess P., Harris M. (2019). Disordered eating among Australian adolescents: Prevalence, functioning, and help received. Int. J. Eat. Disord..

[6] Merikangas K.R., He J.-P., Burstein M., Swanson S.A., Avenevoli S., Cui L., Benjet C., Georgiades K., Swendsen J. (2010). Lifetime prevalence of mental disorders in US adolescents: Results from the National Comorbidity Study–Adolescent Supplement (NCS-A). J. Am. Acad. Child Adolesc. Psychiatry.

[7] Götestam K.G., Agras W.S. (1995). General population-based epidemiological study of eating disorders in Norway. Int. J. Eat. Disord..

[8] Correa M.L., Zubarew T., Silva P., Romero M.I. (2006). Prevalencia de riesgo de trastornos alimentarios en adolescentes mujeres escolares de la Región Metropolitana. Rev. Chil. Pediatr..

[9] Villalobos-Hernández A., Bojórquez-Chapela I., Hernández-Serrato M.I., Unikel-Santoncini C. (2023). Prevalencia de conductas alimentarias de riesgo en adolescentes mexicanos: Ensanut Continua 2022. Salud Pública Méx..

[10] Teixeira A.A., Roque M.A., de Freitas A.A., dos Santos N.F., Garcia F.M., Khoury J.M., Albuquerque M.R., das Neves M., Garcia F.D. (2021). Brazilian version of the SCOFF questionnaire for screening eating disorder risk: Cultural adaptation and validation study in a university population. Braz. J. Psychiatry.

[11] Mérida-Pérez C., López-Hartmann R. (2013). Prevalencia de los trastornos de la conducta alimentaria y su relación con la ansiedad y depresión en adolescentes de secundaria de la ciudad de La Paz. Rev. Investig. Psicol..

[12] Zila-Velasque J.P., Grados-Espinoza P., Regalado-Rodríguez K.M., Luna-Córdova C.J., Calderón G.S.S., Díaz-Vargas M., Sifuentes-Rosales J., Diaz-Vélez C. (2025). Prevalence and factors associated with eating disorders in Peruvian Human Medicine students in the context of the COVID-19 pandemic: A multicentre study. Rev. Colomb. Psiquiatr..

[13] Martínez P. (2003). Estudio epidemiológico de los trastornos alimentarios y factores asociados en Lima Metropolitana. Rev. Psicol..

[14] Año-Flores K.L., Arenas-Yuca K.E., Franco-Phuyo L., Tacuri-Figueroa B. (2019). Factores de riesgo asociados a los trastornos de la conducta alimentaria en estudiantes de ciencias de la salud de la Universidad Andina del Cusco, 2019. Yachay Rev. Cienc. Cult..

[15] Kong-Lozano L.A., Villanueva-Salazar H.A., Díaz-Ortega J.L. (2021). Relación entre riesgo de trastorno de conducta alimentaria y composición corporal en estudiantes, Trujillo, Perú. Rev. Cient. Cienc. Salud.

[16] Laghi F., McPhie M.L., Baumgartner E., Rawana J.S., Pompili S., Baiocco R. (2016). Family functioning and dysfunctional eating among Italian adolescents: The moderating role of gender. Child Psychiatry Hum. Dev..

[17] Öztürk A., Limnili G., Kartal M. (2022). Association between eating disorder risk and family structure and social appearance anxiety among college freshmen. Alpha Psychiatry.

[18] Amiri S., Sabzehparvar M. (2025). Childhood maltreatment and the risk of eating disorders: A meta-analysis of observational studies. Neuropsychiatrie.

[19] Mateos-Agut M., García-Alonso I., De La Gándara-Martín J.J., I Vegas-Miguel M., Sebastián-Vega C., Sanz-Cid B., Martínez-Villares A., Martín-Martínez E. (2014). Family structure and eating behavior disorders. Actas Esp. Psiquiatr..

[20] Berge J.M., Wall M., Larson N., Eisenberg M.E., Loth K.A., Neumark-Sztainer D. (2014). The unique and additive associations of family functioning and parenting practices with disordered eating behaviors in diverse adolescents. J. Behav. Med..

[21] Quiñones J.C.G., Méndez S.P.M., Gutiérrez S.P.C., Tafur Y.M.E., Torres S.J.A., Ortegón D.F.C., Fonseca V.E.P., Ramírez J.A.P. (2023). Relación entre trastornos alimentarios y familia e ideación suicida en adolescentes escolarizadas de Bogotá. Rev. Salud Pública.

[22] González-Quiñones J.C., De la Hoz-Restrepo F. (2011). Relaciones entre los comportamientos de riesgo psicosociales y la familia en adolescentes de Suba, Bogotá. Rev. Salud Pública.

[23] García-Galicia A., Montiel-Jarquín Á.J., Rivera-Zúñiga B.P., Torres-Santiago D., Aréchiga-Santamaría A., González-López A.M., López-Bernal C.A. (2021). Trastornos alimentarios en menores de 5 años y su relación con la funcionalidad familiar. Rev. Fac. Med. Hum..

[24] Morales-Pernalete A.R., Gordillo-Gutierrez C.A., Pérez-Alvarado C.J., Marcano-Flores D.A., Pérez-Pérez F.A., Flores-Navas H.L., Pérez Navea J.M., Pérez Linarez M.A., Meléndez Flores P.M. (2014). Factores de riesgo para los trastornos por atracón y su asociación con la obesidad en adolescentes. Gac. Med. Mex..

[25] Vásquez-Becerra D.G. (2018). Disfunción familiar como factor asociado a trastorno de la conducta alimentaria en estudiantes. Tesis de Licenciatura en Medicina.

[26] Dahill L.M., Morrison N.M.V., Mannan H., Mitchison D., Touyz S., Bussey K., Trompeter N., Hay P. (2022). Exploring associations between positive and negative valenced parental comments about adolescents’ bodies and eating and eating problems: A community study. J. Eat. Disord..

[27] Balottin L., Mannarini S., Mensi M.M., Chiappedi M., Gatta M. (2017). Triadic interactions in families of adolescents with anorexia nervosa and families of adolescents with internalizing disorders. Front. Psychol..

[28] Langdon-Daly J., Serpell L. (2017). Protective factors against disordered eating in family systems: A systematic review of research. J. Eat. Disord..

[29] Lima-Rodríguez J.S., Lima-Serrano M., Domínguez-Sánchez I. (2015). Psychometric properties of an instrument to measure family disease management. Int. J. Clin. Health Psychol..

[30] Solmi F., Hatch S.L., Hotopf M., Treasure J., Micali N. (2015). Validation of the SCOFF questionnaire for eating disorders in a multiethnic general population sample. Int. J. Eat. Disord..

[31] Rueda-Jaimes G.E., Díaz-Martínez L.A., Ortiz-Barajas D.P., Pinzón-Plata C., Rodríguez-Martínez J., Cadena-Afanador L.P. (2005). Validación del cuestionario SCOFF para el cribado de trastornos de la conducta alimentaria en adolescentes escolares. Aten. Primaria.

[32] Campo-Arias A., Caballero-Domínguez C.C. (2021). Confirmatory factor analysis of the family APGAR questionnaire. Rev. Colomb. Psiquiatr. (Engl. Ed.).

[33] Zeladita-Huaman J.A., Zegarra-Chapoñan R., Cuba-Sancho J.M., Castillo-Parra H., Chero-Pacheco V.H., Morán-Paredes G.I. (2022). Validation of a Bullying Scale in Peruvian Adolescents and Gender-Specific Differences. Int. J. Psychol. Res..

[34] Sinclair S.J., Blais M.A., Gansler D.A., Sandberg E., Bistis K., LoCicero A. (2010). Psychometric properties of the Rosenberg Self-Esteem Scale: Overall and across demographic groups living within the United States. Eval. Health Prof..

[35] Ventura-León J., Caycho-Rodríguez T., Barboza-Palomino M., Salas G. (2018). Evidencias psicométricas de la escala de autoestima de Rosenberg en adolescentes limeños. Rev. Interam. Psicol..

[36] Alaniz G., García-Meda M., Moreno C., Ortega J., Morales M., Romo L. (2023). Estudio de validación de la escala de autoestima de Rosenberg en población adolescente de educación pública en Jalisco. Lat. Am. Rev. Cienc. Soc. Humanid..

[37] Cheng C., Dong D., He J., Zhong X., Yao S. (2020). Psychometric properties of the 10-item Connor–Davidson Resilience Scale (CD-RISC-10) in Chinese undergraduates and depressive patients. J. Affect. Disord..

[38] Lima-Sánchez D.N., Navarro-Escalera A., Fouilloux-Morales C., Tafoya-Ramos S.A., Campos-Castolo E.M. (2020). Validation of the 10-item resilience scale with Mexican college students. Rev. Med. Inst. Mex. Seguro Soc..

[39] Álvarez-García H.B., Lugo-González I.V., González-Betanzos F. (2023). Psychometric properties of the Insomnia Severity Index (ISI) in Mexican adults. Interacciones.

[40] González-Rivera J.A., Pagán-Torres O.M., Pérez-Torres E.M. (2020). Depression, Anxiety and Stress Scales (DASS-21): Construct validity problem in Hispanics. Eur. J. Investig. Health Psychol. Educ..

[41] Antúnez Z., Vinet E.V. (2012). Escalas de Depresión, Ansiedad y Estrés (DASS-21): Validación de la versión abreviada en estudiantes universitarios chilenos. Ter. Psicol..

[42] Caldera-Zamora I.A., Martín-del-Campo-Rayas P., Caldera-Montes J.F., Reynoso-González O.U., Zamora-Betancourt M.R. (2019). Predictores de conductas alimentarias de riesgo en estudiantes de bachillerato. Rev. Mex. Trastor. Aliment..

[43] Marmo J. (2014). Estilos parentales y factores de riesgo asociados a la patología alimentaria. Av. Psicol..

[44] Croll J., Neumark-Sztainer D., Story M., Ireland M. (2002). Prevalence and risk and protective factors related to disordered eating behaviors among adolescents: Relationship to gender and ethnicity. J. Adolesc. Health.

[45] Esteves-Villanueva A.R., Paz-Paredes-Mamani R., Calcina-Condori C.R., Yapuchura-Saico C.R. (2020). Habilidades sociales en adolescentes y funcionalidad familiar. Comunicacción.

[46] Valenzuela-Mujica M.T., Ibarra R.A., Zubarew G.T., Correa M.L. (2013). Prevención de conductas de riesgo en el adolescente: Rol de familia. Index Enferm..

[47] Salas-Ramos H.P. (2017). Relación Entre Hábitos Alimentarios y Riesgo de Trastornos de Conducta Alimentaria en Adolescentes de Secundaria de una Institución Educativa Estatal, Los Olivos 2015. Bachelor’s Thesis.

[48] Díaz R.F., Pilicita S.L., Godoy V.L., Donoso F.A. (2022). Trastornos de conducta alimentaria grave en adolescentes durante la pandemia COVID-19: Un llamado a la acción. Andes Pediatr..

[49] EsSalud (2021). EsSalud Alerta Sobre Aumento de Casos de Trastornos Alimentarios en Adolescentes Desde los 11 Años.

[50] Galiano Ramírez M.C., Prado Rodríguez R.F., Mustelier Bécquer R. (2021). Salud mental en la infancia y adolescencia durante la pandemia de COVID-19. Rev. Cubana Pediatr..

[51] Lazo-Montoya Y., Quenaya A., Mayta-Tristán P. (2015). Influencia de los medios de comunicación y el riesgo de padecer trastornos de la conducta alimentaria en escolares mujeres en Lima, Perú. Arch. Argent. Pediatr..

[52] García-Puertas D. (2020). Influencia del uso de Instagram sobre la conducta alimentaria y trastornos emocionales: Revisión sistemática. Rev. Esp. Comun. Salud.

[53] Arévalo H., Urina-Triana M., Santacruz J.C. (2020). Impacto del aislamiento preventivo obligatorio en la actividad física diaria y en el peso de los niños durante la pandemia por SARS-CoV-2. Rev. Colomb. Cardiol..

[54] Piñeros-Ortíz S., Molano-Caro J., López-de-Mesa C. (2010). Factores de riesgo de los trastornos de la conducta alimentaria en jóvenes escolarizados en Cundinamarca (Colombia). Rev. Colomb. Psiquiatr..

[55] Portela-de-Santana M.L., da Costa Ribeiro-Junior H., Mora-Giral M., Raich R.M. (2012). La epidemiología y los factores de riesgo de los trastornos alimentarios en la adolescencia: Una revisión. Nutr. Hosp..

[56] Veses A.M., Martínez-Gómez D., Gómez-Martínez S., Vicente-Rodriguez G., Castillo R., Ortega F.B., González-Gross M., Calle M.E., Veiga O.L., Marcos A. (2014). Physical fitness, overweight and the risk of eating disorders in adolescents: The AVENA and AFINOS studies. Pediatr. Obes..

[57] Kokka I., Mourikis I., Bacopoulou F. (2023). Psychiatric disorders and obesity in childhood and adolescence: A systematic review of cross-sectional studies. Children.

[58] Jáuregui-Lobera I. (2011). Sobrepeso y obesidad como factores de riesgo de los trastornos de la conducta alimentaria. Med. Clin..

[59] Ruíz-Martínez A.O., Vázquez-Arévalo R., Mancilla-Díaz J.M., Viladrich-i-Segués C., Halley-Castillo M.E. (2013). Factores familiares asociados a los trastornos alimentarios: Una revisión. Rev. Mex. Trastor. Aliment..

[60] Silberg J.L., Bulik C.M. (2005). The developmental association between eating disorders symptoms and symptoms of depression and anxiety in juvenile twin girls. J. Child Psychol. Psychiatry.

[61] McCabe M.P., Ricciardelli L.A. (2005). A prospective study of pressures from parents, peers, and the media on extreme weight change behaviors among adolescent boys and girls. Behav. Res. Ther..

[62] Mellor D., McCabe M., Ricciardelli L., Merino M.E. (2008). Body dissatisfaction and body change behaviors in Chile: The role of sociocultural factors. Body Image.

[63] Cortés-Romero C.E., Escobar-Noriega A., Cebada-Ruiz J., Soto-Rodríguez G., Bilbao-Reboredo T., Vélez-Pliego M. (2018). Estrés y cortisol: Implicaciones en la ingesta de alimento. Rev. Cuba. Invest. Bioméd..

[64] Hudson J.I., Hiripi E., Pope H.G., Kessler R.C. (2007). The prevalence and correlates of eating disorders in the National Comorbidity Survey Replication. Biol. Psychiatry.

[65] Holderness C.C., Brooks-Gunn J., Warren M.P. (1994). Co-morbidity of eating disorders and substance abuse: Review of the literature. Int. J. Eat. Disord..

[66] Moreno-Otero R., Cortés J.R. (2008). Nutrición y alcoholismo crónico. Nutr. Hosp..

[67] Schneider J., Pegram G., Gibson B., Talamonti D., Tinoco A., Craddock N., Matheson E., Forshaw M. (2023). A mixed-studies systematic review of the experiences of body image, disordered eating, and eating disorders during the COVID-19 pandemic. Int. J. Eat. Disord..

[68] Huete-Córdova M.A. (2022). Trastorno de conducta alimentaria durante la pandemia del SARS-CoV-2. Rev. Neuropsiquiatr..

[69] Pengcheng L., Longfei Z., Shujuan C., Xiaojie W. (2021). Association between household overcrowding and depressive mood among Chinese residents. J. Affect. Disord..

[70] Calderón M.A., Moreno C.P., Rojas C.D., Barboza-del-C J. (2005). Consumo de alimentos según condición de pobreza en mujeres en edad fértil y niños de 12 a 35 meses de edad. Rev. Peru. Med. Exp. Salud Pública.

[71] Calvete E., Las-Hayas C., Gómez-Del-Barrio A. (2018). Longitudinal associations between resilience and quality of life in eating disorders. Psychiatry Res..

[72] Hayas C.L., Padierna J.A., Muñoz P., Aguirre M., del Barrio A.G., Beato-Fernández L., Calvete E. (2016). Resilience in eating disorders: A qualitative study. Women Health.

[73] Seigel K., Broman J.E., Hetta J. (2004). Problemas de sueño y síntomas de trastornos de la conducta alimentaria en mujeres jóvenes. Eur. J. Psychiat. (Ed. Esp.).

[74] Hernández-Ruiz-de-Eguilaz M., Martínez-de-Morentin-Aldabe B., Almiron-Roig E., Pérez-Diez S., San-Cristóbal-Blanco R., Navas-Carretero S., Martínez J.A. (2018). Influencia multisensorial sobre la conducta alimentaria: Ingesta hedónica. Endocrinol. Diabetes Nutr. (Engl. Ed.).

[75] Forero-Bogotá M.A., Gómez-Leguizamón M. (2021). Determinantes fisiológicos y ambientales de la regulación del control de la ingesta de alimentos. Rev. Nutr. Clin. Metab..

[76] Raney T., Thornton L.M., Berrettini W., Brandt H., Crawford S., Fichter M.M., Halmi K.A., Johnson C., Kaplan A.S., LaVia M. (2008). Influence of overanxious disorder of childhood on the expression of anorexia nervosa. Int. J. Eat. Disord..

[77] Fernández-Ruiz M., Masjuan N., Costa-Ball D., Cracco C. (2015). Funcionamiento familiar y trastornos de la conducta alimentaria: Una investigación desde el modelo circumplejo. Cienc. Psicológicas.

[78] Sepúlveda A.R., Moreno A., Beltrán L. (2020). Actualización de las intervenciones dirigidas al contexto familiar en los trastornos del comportamiento alimentario: El rol de los padres. Rev. Psicoter..

[79] Coop A., Clark A., Morgan J., Reid F., Lacey J.H. (2024). The use and misuse of the SCOFF screening measure over two decades: A systematic literature review. Eat. Weight Disord..

[80] Ordóñez Azuara Y., Gutiérrez Herrera R.F., Méndez Espinoza E., Alvarez Villalobos N.A., Lopez Mata D., de la Cruz de la Cruz C. (2020). Association of family typology and dysfunction in families with adolescents from a Mexican population. Aten. Primaria.

[81] Quiles-Marcos Y., Quiles-Sebastián M.J., Pamies-Aubalat L., Botella-Ausina J., Treasure J. (2013). Peer and family influence in eating disorders: A meta-analysis. Eur. Psychiatry.

