# Supplementary material for: Association Between Family Dysfunction and Risk for Eating Disorders in Adolescents
Source: J Clin Med. 2026 Feb 25;15(5):1726. doi: 10.3390/jcm15051726 (PMC12985996; doi:10.3390/jcm15051726)
Supplement: Supplementary file 1 [file jcm-15-01726-s001.zip › jcm-3968814-supplementary.pdf]

Description of measurement_instruments

1. Bullying – EBIPQ (European Bullying Intervention Project Questionnaire)

   School bullying was assessed with a validated self-report questionnaire that distinguishes two roles: victimization and aggression. It consists of 14 items (7 for victimization and 7 for aggression), each with a 5-point Likert scale ranging from 0 = "never" to 4 = "always," referring to the frequency of occurrence within a recent specific period. The subscale scores range from 0 to 28. In Peruvian studies, the presence of bullying has been defined when the score in any subscale is ≥7, indicating that the participant has been either a victim or a perpetrator of bullying behaviors at least several times [1]. Regarding its psychometric properties, a study conducted in Peru with 532 school students reported good validity and reliability: the study found a factorial structure similar to the original instrument through exploratory factor analysis, explaining around 48.6% of the variance, and internal reliabilities (Cronbach's alpha) of α_total = 0.856, α_victimization = 0.807, and α_aggression = 0.828 [1].

2. Self-esteem – Rosenberg Self-Esteem Scale

   A standardized 10-item instrument with 4-point Likert-type responses (1 = strongly disagree to 4 = strongly agree), five formulated in a positive direction and five in a negative direction (the latter are reverse-scored). It provides a total score of 10–40 (higher score = higher self-esteem) and is primarily conceived as unidimensional (global self-esteem), although the literature acknowledges method effects from positive/negative wording and bifactor models (positive/negative self-esteem). In this study, for descriptive purposes, we followed categories commonly used in Spanish-language research: low (≤25), medium (26–29), and high (30–40); these cut-off points are practical and not part of the original scale, and therefore may vary depending on the population [2]. The scale, originally designed for high school students, has shown construct validity and internal consistency in Latin American adolescents. In Peru, studies with school samples (n = 450 and n = 481) confirmed good exploratory factor analysis fit, sex invariance, and adequate reliability (H coefficients > .80) [3]. In Mexico, a study with adolescents (n = 302) reported composite reliability = 0.85 and a bidimensional structure in EFA with adequate fit, supporting its use in school settings [4].

3. Resilience – CD-RISC short version

   The abbreviated version of this scale consists of 10 items that assess global resilience as the ability to cope with adversity and recover from stressful experiences. Each item is answered on a 5-point Likert scale (0 = never, 1 = rarely, 2 = sometimes, 3 = often, 4 = almost always), with a total score ranging from 0 to 40, where higher values indicate greater resilience. The scale is essentially considered unidimensional, without rigid diagnostic categories; however, in practice, normative ranges of low resilience (≤20), moderate resilience (21–30), and high resilience (31–40) have been used to facilitate interpretation in applied studies [5]. In Mexico, a validation study conducted among 1,333 first-year medical students (mean age = 18 years; range = 17–39) confirmed the unidimensional structure of the instrument through factor analysis, with one factor explaining 96.2% of the total variance. The scale showed excellent internal reliability (Cronbach's α = 0.935) and adequate temporal stability (test-retest $r = 0.521$; $p = 0.01$). As evidence of validity, the instrument showed moderate convergent validity against a culturally validated resilience scale developed in Mexico ($r = 0.61$; $p < 0.001$) and divergent validity through negative correlations with the Beck Anxiety Inventory ($r = -0.214$; $p < 0.05$) and the Beck Depression Inventory ($r = -0.199$; $p < 0.05$) [6].

4. Insomnia – ISI (Insomnia Severity Index / adolescent version)

   This is a brief standardized questionnaire that assesses the subjective perception of insomnia severity and its impact on daily life. It consists of 7 items (the original version; some adaptations extend up to 13 items in clinical contexts), each with a 5-point Likert scale (0 = none; 4 = extremely). The items cover core dimensions: difficulty falling asleep, difficulty staying asleep, early morning awakenings, satisfaction with sleep pattern, interference with daily functioning, perception of noticeable impairment, and concern about the sleep problem. The total score ranges from 0 to 28, classified into four severity categories: 0–7 (no clinically significant insomnia), 8–14 (subthreshold insomnia), 15–21 (moderate insomnia), and 22–28

(severe insomnia). In Mexico, a recent study with 310 adults compared factorial models and found that the best solution was a bifactor model, with a general severity factor and a specific impact factor, showing excellent fit indices (CFI = 0.98; TLI = 0.96; RMSEA = 0.07; SRMR = 0.03). Internal reliability was adequate, with ω general = 0.86 and ω impact = 0.86, and factorial invariance by sex was confirmed. In addition, the ISI demonstrated convergent validity through high correlations with the Athens Insomnia Scale (r = 0.84) and divergent validity by correlating weakly to moderately with the Epworth Sleepiness Scale (r = 0.39–0.44) [7].

5. Depression, Anxiety, and Stress Scale – 21 items (DASS-21)

This is the abbreviated version of the original 42-item scale developed by Lovibond and Lovibond. It consists of 21 items distributed across three dimensions—depression, anxiety, and stress—with seven items in each subscale, answered on a four-point Likert scale (0 = did not apply to me at all to 3 = applied to me very much or most of the time). Each subscale yields a score between 0 and 21. For clinical interpretation, it is recommended to multiply the scores by two to equate them with the DASS-42, with severity categories that classify symptoms as normal, mild, moderate, severe, or extremely severe depending on the construct evaluated. In psychometric terms, the DASS-21 has consistently shown high internal consistency, with Cronbach's alpha values above 0.80 across different populations [8]. In Chile, a validation study with 484 university students confirmed, through exploratory factor analysis, a three-factor structure explaining 49.99% of the variance. It also demonstrated adequate convergent and divergent validity, showing significant correlations with the Beck Depression Inventory (BDI-II), the Beck Anxiety Inventory (BAI), and the SCL-90-R. Internal reliability for each subscale was satisfactory, with α values ranging from 0.82 to 0.86, supporting that the DASS-21 is a brief, valid, and reliable instrument for assessing symptoms of depression, anxiety, and stress in Latin American youth [9].

References

1. Zeladita-Huaman, J.A.; Zegarra-Chapoñan, R.; Cuba-Sancho, J.M.; Castillo-Parra, H.; Chero-Pacheco, V.H.; Morán-Paredes, G.I. Validation of a Bullying Scale in Peruvian Adolescents and Gender-Specific Differences. *Int. J. Psychol. Res. (Medellín)* **2022**, *15*(2), 105–113. https://doi.org/10.21500/20112084.5522
2. Sinclair, S.J.; Blais, M.A.; Gansler, D.A.; Sandberg, E.; Bistis, K.; LoCicero, A. Psychometric properties of the Rosenberg Self-Esteem Scale: Overall and across demographic groups living within the United States. *Eval. Health Prof.* **2010**, *33*(1), 56–80. https://doi.org/10.1177/0163278709356187
3. Ventura-León, J.; Caycho-Rodríguez, T.; Barboza-Palomino, M.; Salas, G. Evidencias psicométricas de la escala de autoestima de Rosenberg en adolescentes limeños. *Rev. Interam. Psicol.* **2018**, *52*(1). https://doi.org/10.30849/rip/ijp.v52i1.363
4. Alaniz, G.; García-Meda, M.; Moreno, C.; Ortega, J.; Morales, M.; Romo, L. Estudio de validación de la escala de autoestima de Rosenberg en población adolescente de educación pública en Jalisco. *Lat. Am. Rev. Cienc. Soc. Humanid.* **2023**, *4*(6), [sin páginas]. https://doi.org/10.56712/latam.v4i6.1437
5. Cheng, C.; Dong, D.; He, J.; Zhong, X.; Yao, S. Psychometric properties of the 10-item Connor–Davidson Resilience Scale (CD-RISC-10) in Chinese undergraduates and depressive patients. *J. Affect. Disord.* **2020**, *260*, 412–419. https://doi.org/10.1016/j.jad.2019.10.018
6. Lima-Sánchez, D.N.; Navarro-Escalera, A.; Fouilloux-Morales, C.; Tafoya-Ramos, S.A.; Campos-Castolo, E.M. Validation of the 10-item resilience scale with Mexican college students. *Rev. Med. Inst. Mex. Seguro Soc.* **2020**, *58*(3), 292–297. https://doi.org/10.24875/RMIMSS.M21000033
7. Álvarez-García, H.B.; Lugo-González, I.V.; González-Betanzos, F. Psychometric properties of the Insomnia Severity Index (ISI) in Mexican adults. *Arch.* **2023**, *9*, 1–12. https://doi.org/10.24016/2023.v9.311
8. González-Rivera, J.A.; Pagán-Torres, O.M.; Pérez-Torres, E.M. Depression, Anxiety and Stress Scales (DASS-21): Construct validity problem in Hispanics. *Eur. J. Investig. Health Psychol. Educ.* **2020**, *10*(1), 375–389. https://doi.org/10.3390/ejihpe10010028
9. Antúnez, Z.; Vinet, E.V. Escalas de Depresión, Ansiedad y Estrés (DASS-21): Validación de la versión abreviada en estudiantes universitarios chilenos. *Ter. Psicol.* **2012**, *30*(3), 49–55. https://doi.org/10.4067/S0718-48082012000300005
